# Effects of Extended Postmortem Interval on Microbial Communities in Organs of the Human Cadaver

**DOI:** 10.3389/fmicb.2020.569630

**Published:** 2020-12-08

**Authors:** Holly Lutz, Alexandria Vangelatos, Neil Gottel, Antonio Osculati, Silvia Visona, Sheree J. Finley, Jack A. Gilbert, Gulnaz T. Javan

**Affiliations:** ^1^Department of Pediatrics, University of California, San Diego, La Jolla, CA, United States; ^2^Scripps Institution of Oceanography, University of California, San Diego, La Jolla, CA, United States; ^3^Biological Sciences Division, University of Chicago, Chicago, IL, United States; ^4^Department of Public Health, Experimental and Forensic Medicine, University of Pavia, Pavia, Italy; ^5^Physical Sciences Department, Forensic Science Programs, Alabama State University, Montgomery, AL, United States

**Keywords:** thanatomicrobiome, cadaver, 16S rRNA, internal organs, manner of death

## Abstract

Human thanatomicrobiota studies have shown that microorganisms inhabit and proliferate externally and internally throughout the body and are the primary mediators of putrefaction after death. Yet little is known about the source and diversity of the thanatomicrobiome or the underlying factors leading to delayed decomposition exhibited by reproductive organs. The use of the V4 hypervariable region of bacterial 16S rRNA gene sequences for taxonomic classification (“barcoding”) and phylogenetic analyses of human postmortem microbiota has recently emerged as a possible tool in forensic microbiology. The goal of this study was to apply a 16S rRNA barcoding approach to investigate variation among different organs, as well as the extent to which microbial associations among different body organs in human cadavers can be used to predict forensically important determinations, such as cause and time of death. We assessed microbiota of organ tissues including brain, heart, liver, spleen, prostate, and uterus collected at autopsy from criminal casework of 40 Italian cadavers with times of death ranging from 24 to 432 h. Both the uterus and prostate had a significantly higher alpha diversity compared to other anatomical sites, and exhibited a significantly different microbial community composition from non-reproductive organs, which we found to be dominated by the bacterial orders MLE1-12, Saprospirales, and Burkholderiales. In contrast, reproductive organs were dominated by Clostridiales, Lactobacillales, and showed a marked decrease in relative abundance of MLE1-12. These results provide insight into the observation that the uterus and prostate are the last internal organs to decay during human decomposition. We conclude that distinct community profiles of reproductive versus non-reproductive organs may help guide the application of forensic microbiology tools to investigations of human cadavers.

## Introduction

During life, the human microbiome serves important health-related functions including nutrient acquisition, pathogen defense, energy salvage, and immune defense training (Gilbert et al., 2016). The microbiome has also been linked to cardiovascular, metabolic, and immune diseases, as well as mental health disorders via the gut-brain-axis (GBA; Gilbert et al., 2018). The microbiota GBA is a complex, bidirectional circuit that links the neural, endocrine, and immunological systems with the microbial communities in the gut (Grenham et al., 2011; Cryan and Dinan, 2012; Meckel and Kiraly, 2019). After death, microbial communities present within and on the body are exposed to radical environmental changes, and recent studies have shown that microbial succession among mammalian cadavers follows a metabolically predictable progression (Metcalf et al., 2016; Javan et al., 2019).

Forensic microbiology is an emerging field of study in which microorganisms serve as forensic tools to potentially address unanswered forensic questions such as cause and time of death. The detection of pathogenic microbes has evidential value in medicolegal evaluations pertaining to etiological determinations of pathological death. However, the connection between postmortem microbial communities and violent death (homicide, suicide, and overdoses) is still in its infancy. Postmortem microbial communities’ succession related to human remains has been proven to be predictable and applicable for forensic science and criminal investigations (Damann et al., 2015; DeBruyn and Hauther, 2017; Pechal et al., 2018). By determining the intricate relationships between microbial abundances in specific organs, thanatomicrobiome (microbiome of death) studies have the potential to resolve knowledge gaps in a death investigation that could match specific taxa to a point on a “microbial clock” in a regression model (Metcalf, 2019). Advances in DNA sequencing technologies paired with increased understanding of the human postmortem microbiome have hinted at the possibility that the cadre of microbes could be used as a predominant drivers of decay (Metcalf et al., 2016; Buresova et al., 2019) and as trace evidence to link distinct people to objects with which they have previously interacted (Fierer et al., 2010; Lax et al., 2015; Park et al., 2017; Schmedes et al., 2017; Kodama et al., 2019). Recent studies have also shown that the microbiome may be used to estimate the amount of time that has elapsed since death, referred to as the postmortem interval (PMI), allowing investigators to establish a potential timeline of death (Maujean et al., 2013; Pechal et al., 2014; Cobaugh et al., 2015; Hauther et al., 2015; Burcham et al., 2016; Javan et al., 2016; Metcalf et al., 2016; Javan et al., 2017).

The microbial composition and abundance associated with internal organ tissues are dependent on temperature, and PMI, since bacteria have different growth optima based on the physicochemical constraints of their environment (Deutscher, 2008; Ercolini et al., 2009; Rakoff-Nahoum et al., 2016). Also, microbial abundances associated with the body antemortem may play a role in decay, as a cadaver of an aged adult human, with approximately 40 trillion microbial cells, decays more rapidly than a deceased fetus or newborn (Campobasso et al., 2001), which usually has reduced microbial colonization density (Dunn et al., 2017). Of course, as suggested by Campobasso et al. (2001), these trends are contingent upon the medications and disease state of the individual prior to death. In Italy, as with other localities in the world, the determination of the cause of death is essential for legal documentation and public health in regard to epidemiological surveillance. For example, in the United States, the causes of death are classified as accident, homicide, natural, suicide, and undetermined. In contrast to United States death certificates, Italian death certificates do not offer an undetermined cause of death. Death investigation systems serve as conduits for epidemiological data collection and as an important sentinel system in the event of mass pandemics of fatal diseases as in the recent cases of novel coronavirus (severe acute respiratory syndrome coronavirus-2, SARS-CoV-2, or COVID-19).

The cause of death is used to clarify the circumstances surrounding how a person died or how an injury was sustained. Here we focus on Italian cadavers due to their extended PMIs (up to 24–432 h) compared to those of other geographic locations (e.g., the United States). There was an even spread of PMIs: 22 cases with PMIs less than 72 h and 18 with PMIs 72–432 h. The Italian Regolamento di Polizia Mortuaria, Law number 285, Article 8 of 1990 prohibits an autopsy prior to 24 h of discovering a dead body (Frati et al., 2006). In the current study, we hypothesized that microbial taxa composition in postmortem internal organ tissues are dependent on temporal (PMI) and forensic (cause of death) influences. To test this hypothesis, the V4 hypervariable region of the 16S rRNA gene was probed to investigate the extent to which microbial associations among different body organs in human cadavers can be used to predict the cause of death and/or PMI. By sampling different anatomical body sites of human cadavers with various causes of death (accident, natural, suicide, and homicide), we were able to ascertain that cause of death has a significant influence on microbial community composition of postmortem tissues, and that despite these differences, commonalities may still be identified among tissues.

## Materials and Methods

### Collection of Autopsy Specimens

Sequencing of amplicons of the 16S rRNA V4 hypervariable region was performed using microbial DNA from postmortem organ tissue from 40 Italian cadavers including brain, heart, liver, spleen, prostate, and uterus collected at autopsy from criminal casework cadavers. All cadaver samples were collected before the COVID-19 outbreak in Wuhan, China; therefore, COVID-19 specimens were not a part of this study. Careful precautions were taken to reduce contamination and maintain hygienic laboratory conditions. Bodies were kept in the morgue at 1°C at the Department of Public Health, Experimental and Forensic Medicine at the University of Pavia in Pavia, Italy (Supplementary Table 1). The study included 14 females and 26 male cadavers, and the youngest case was in the range 15–20 and the oldest 85–90. All PMIs were confirmed by official Daily Crime Logs with the shortest PMI was 24 h and the longest was 18 days. Cadavers were categorized into four groups according to cause of death: accidental, homicide, natural, and suicide. Autopsies were performed in the morgue with ambient temperatures between 8 and 10°C. Postmortem samples were uniformly excised from an identical section of each tissue of the brain, heart, liver, spleen, prostate, and uterus of each cadaver. For example, cardiac tissue was homogeneously removed from the left ventricle area of the hearts. During autopsies, tissues that were extracted from internal organs were placed into labeled sterile polyethylene bags. After collection, samples were transported on dry ice to the Thanatos Laboratory, a core facility on the campus of Alabama State University in Montgomery, AL, United States, and stored at −80°C until further analysis. Using sterile, disposable surgical scalpels, a representative sample of 40–50 mg of tissue was aliquoted from each organ for microbial analyses. Tissues were collected using protocols approved by Alabama State University’s Institutional Review Board (2018400) in accordance with Italian laws regarding personal data treatment. The reference Italian law is the authorization n9/2016 of the guarantor of privacy. This authorization was then replaced by Regulation (EU) 2016/679 of the European Parliament and of the Council. The specimens were anonymized before transferring to the Thanatos Lab at Alabama State University. In cases of deceased subjects, consent is not required, as the samples were taken for clinical/forensic purposes and because it is not possible to contact the next of kin in anonymized circumstances.

### DNA Extraction and Sequencing

DNA was extracted from internal organs using conventional chemical and physical disruption protocols (Urakawa et al., 2010) using the phenol chloroform method, which is specifically optimized for recovery of microbial DNA from low-yield, highly decayed postmortem samples (Can et al., 2014). We used the standard 515F and 806R primers (Caporaso et al., 2011, 2012; Kozich et al., 2013) to amplify the V4 region of the 16S rRNA gene, using mitochondrial blockers to reduce amplification of host mitochondrial DNA (*sensu* Lutz et al., 2019). Sequencing was performed using paired-end 150 base reads on an Illumina HiSeq sequencing platform. Following standard demultiplexing and quality filtering using the Quantitative Insights Into Microbial Ecology pipeline (QIIME2; Caporaso et al., 2010) and vsearch8.1 (Rognes et al., 2016). Chimeric sequences were removed and absolute sequence variants (ASVs) were identified using Deblur (Amir et al., 2017), and taxonomy was assigned using the Greengenes Database (May 2013 release).^1^

### Analysis of 16S rRNA Sequencing Data

Following quality filtering and taxonomy assignment, libraries were rarefied to an even read depth of 1000 reads per library; these libraries were used for all subsequent analyses. Alpha diversity was calculated using the Shannon index and measured species richness based on actual observed ASV diversity. Significance of differing mean values for each diversity calculation was determined using the Kruskal–Wallis rank sum test, followed by a *post hoc* Dunn test with Benjamini–Hochberg corrected *p*-values. Two measures of beta diversity (unweighted UniFrac and weighted UniFrac) were calculated using relative abundances of each ASV (calculated as ASV read depth divided by total library read depth). The adonis2 function was used in the R package Vegan v2.4.2 (Oksanen et al., 2018) for PERMAONVA analysis of marginal effects for organ type, sex, age, cause of death, PMI, BMI, with Bonferroni correction for multiple comparisons. In the case of marginal effects of organ type, non-independence of samples was accounted for from the same cadavers by using the strata function available in ADONIS, whereby strata = “case number” was set. Analysis of composition of microbiome (ANCOM) was performed to assess significance of differential abundance based on log2fold change measures between organs and between different causes of death (Mandal et al., 2015). To examine relationships between PMI and the four most abundant bacterial orders, one-way analysis of variance (ANOVA) was performed on independent linear regression models for each bacterial order and human organ. While alpha diversity of each bacterial order (as with alpha diversity of all bacterial taxa) examined exhibited a normal distribution across PMI values [ANOVA, Pr(>*F*) > 0.05], we did identify a significant interaction between organs and PMI [two-way ANOVA, Pr(>*F*) = 0.05]. Additional R packages used for analyses and figure generation included vegan (Oksanen et al., 2018), ggplot2 (Wickham, 2009), and dplyr (Wickham et al., 2018). For a complete list of packages and codes for microbiome analyses.^2^ All 16S rRNA sequence and sample metadata are publicly available via the QIITA platform^3^ under study identifier (ID) 13450 and the European Bioinformatics Institute (EBI) under accession number ERP125301.

## Results

### Sampling and Library Quality

Sampling spanned PMIs of 24–432 h [avg (SD) = 111 (± 93) h] and included tissues from cadavers corresponding to different causes of death grouped into four categories according to each internal organ: accidental (*n* = 51), natural (*n* = 63), homicide (*n* = 13), and suicide (*n* = 31) (Table 1). Following sequence deblurring, quality filtering, and rarefaction a total of 853,850 16S rRNA V4 amplicon sequences comprising 771 ASV reads were generated from 158 sample libraries.

**TABLE 1:** Samples of human cadavers by organ, and cause of death.

	Cause of Death (*n*)				
Organ	Natural	Accident	Homicide	Suicide	Total (*n*)
Female brain	7	7	2	6	22
Male brain	8	3	1	1	13
Female heart	4	6	2	6	18
Male heart	5	4	1	1	11
Female liver	7	8	2	6	23
Male liver	7	4	1	1	13
Female spleen	6	3	1	4	14
Male spleen	6	3	1	0	10
Prostate	5	10	1	5	21
Uterus	8	3	1	1	13
Total	63	51	13	31	158

**n*, number of libraries post-filtering.*

### Alpha and Beta Diversity

**FIGURE 1:**
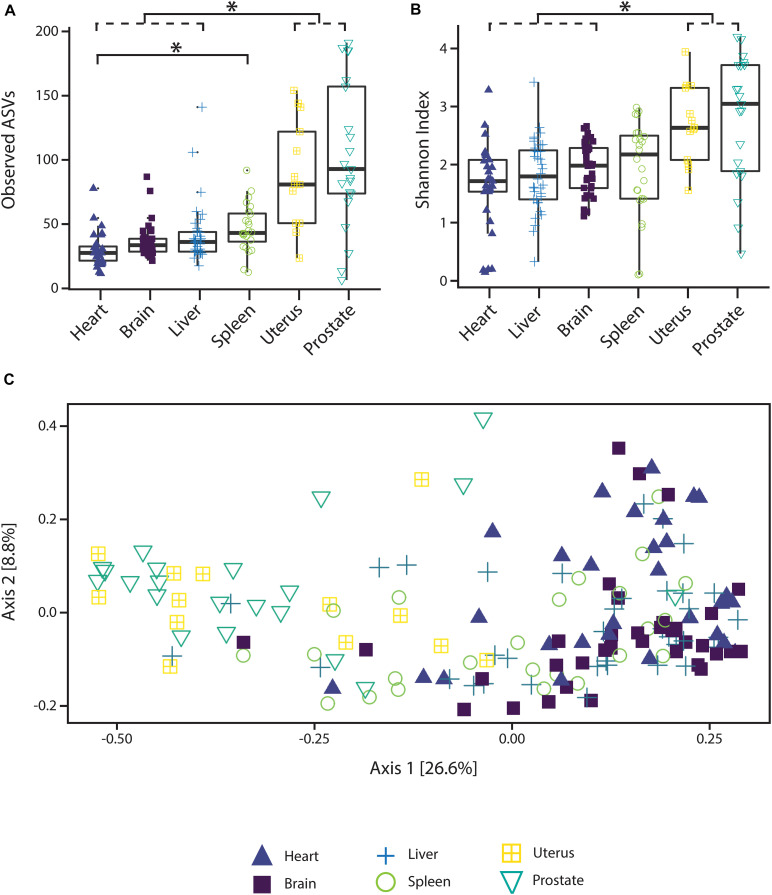
Alpha and beta diversity measures of microbial communities by organ. **(A)** Observed ASV richness by organ, **(B)** Shannon index of microbial richness and evenness by organ. Asterisk indicates *p* < 0.05 Kruskal–Wallis rank sum test with Bonferroni correction. **(C)** PCoA based on unweighted UniFrac measures of microbial dissimilarity by organ.

Alpha diversity varied significantly (*p* < 0.05; Kruskal–Wallis rank sum test with Bonferroni correction) between reproductive organs (prostate and uterus) and non-reproductive organs (brain, heart, and liver, with the exception of spleen) (Figure 1). The two reproductive organs exhibited both higher observed ASV richness values by organ (Figure 1A) and Shannon index of microbial richness and evenness values by organ than non-reproductive organs (Figure 1B).

PERMANOVA analysis of factors contributing to variation among weighted UniFrac beta diversity metrics included organ, sex, PMI, and body mass index (BMI), while factors contributing to variation among unweighted UniFrac beta diversity metrics included organ, age, and cause of death (Table 2). Organ type was the strongest factor contributing to both weighted and unweighted UniFrac beta diversity (*R*^2^ = 0.12 and *R*^2^ = 0.16, respectively) was identified by multidimensional scaling, specifically, principle coordinate analysis (PCoA) analysis (Figure 1C). Although significant, other factors including age, sex, PMI, BMI, and cause of death each explained 1–5% of beta diversity. Differing PERMANOVA results between weighted and unweighted UniFrac for cause of death suggest that specific bacteria may be associated with the cause of death, but that these differences are negligible when taking into account the relative abundance of bacterial taxa; weighted UniFrac measures suggest that although taxonomic composition may not vary significantly between different causes of death the relative abundance of specific taxa vary significantly.

**TABLE 2:** PERMANOVA analysis assessing marginal effects of variables on weighted and unweighted UniFrac beta diversity.

	Df	SumOfSqs	*R*^2^	*F*	*p*-value
**Weighted UniFrac**					
Organ	5	1.03	0.12	4.50	0.001*
Sex	1	0.44	0.05	10.05	0.006*
Age	−	0.09	0.01	2.16	0.270
Cause of death	3	0.24	0.03	1.81	0.186
PMI	−	0.29	0.03	6.61	0.006*
BMI	−	0.19	0.02	4.39	0.018*
**Unweighted UniFrac**					
Organ	5	5.03	0.16	6.00	0.001*
Sex	1	0.31	0.01	2.21	0.102
Age	−	0.39	0.01	2.81	0.042*
Cause of death	3	0.85	0.03	2.02	0.018*
PMI	−	0.41	0.01	2.92	0.048
BMI	−	0.30	0.01	2.17	0.180

*Indicate Bonferroni adjusted *p*-value < 0.05.

### Bacterial Community Composition and Differential Abundance Between Organs and Cause of Death

**FIGURE 2:**
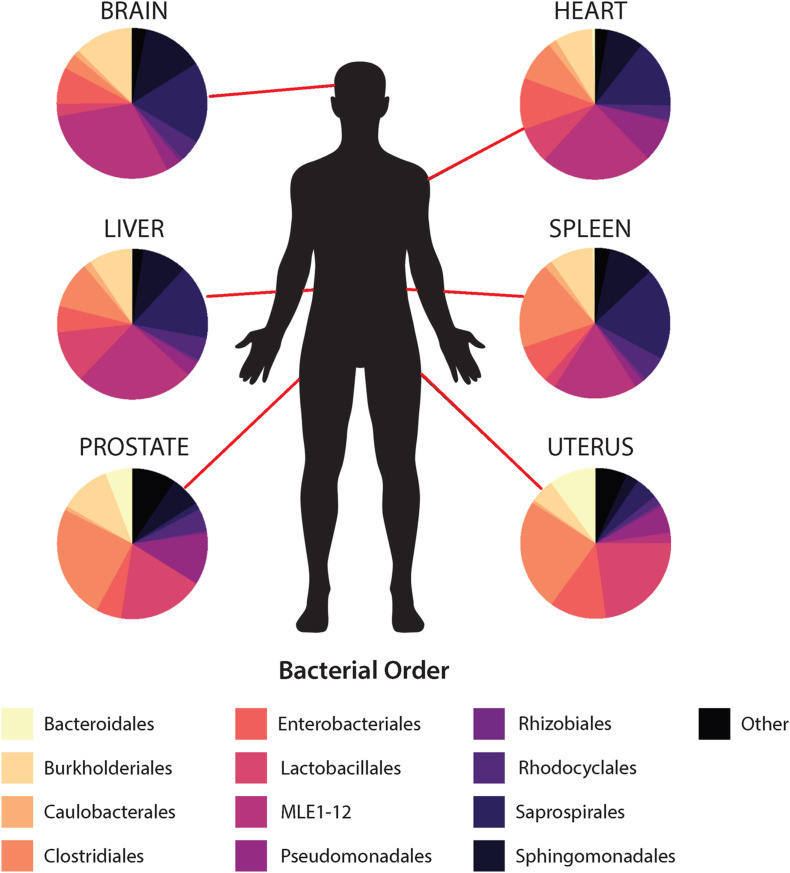
Mean relative abundance of top 10 bacterial orders identified within each organ.

**FIGURE 3:**
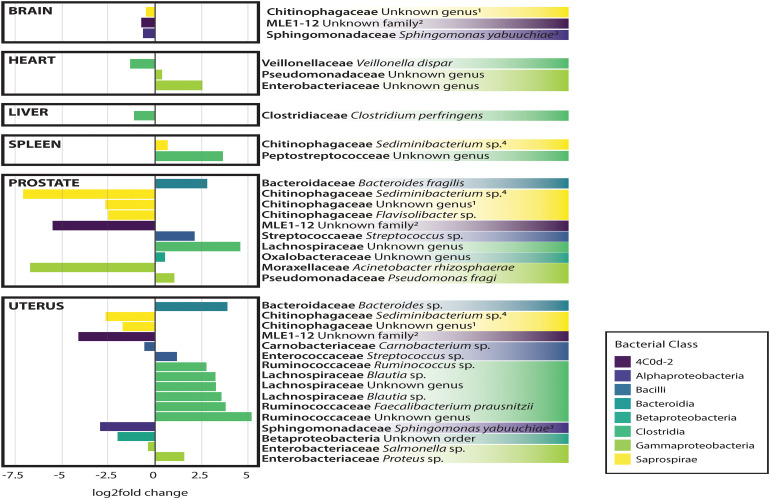
Log2Fold change in differential abundance of individual bacterial OTUs between each organ compared to all other organs. Negative and positive values correspond to under- and over-representation of ASVs in each organ, respectively.

In general, non-reproductive organs were dominated by the bacterial orders MLE1-12 (*Candidatus* Melainabacteria), Saprospirales, and Burkholderiales, while reproductive organs were dominated by Clostridiales, Lactobacillalaes, and showed a marked decrease in relative abundance of MLE1-12 (Figure 2). Analysis of composition of microbiomes between organs confirmed significant differences in relative abundance (measured as the log2fold change in 16S rRNA ASV relative abundance) of multiple bacterial taxa (Figure 3). Specifically, reproductive organs showed significant reduction in a single ASV belonging to the family MLE1-12 and ASVs belonging to the family Chitinophagaceae, including one ASV belonging to the genus *Sediminibacterium*. The prostate also showed a significant reduction in a single ASV in the family Moraxellaceae, belonging to species *Acinetobacter rhizosphaerae*. Both prostate and uterus showed an increased relative abundance of ASVs in the family Bacteroidaceae, identified to species as *Bacteroides fragilis* in the prostate and only as *Bacteroides* sp. in the uterus. A single ASV in the family Streptococcaceae, genus *Streptococcus*, was also found to be increased in both organs. Lastly, both prostate and uterus showed increased relative abundance of ASVs in the family Lachnospiraceae, with one ASV showing an increase in both organs and two other ASVs belonging to the genus *Blautia* showing an increase in the uterus only. Among non-reproductive organs there were no marked patterns. Only three ASVs were identified as being slightly reduced in the brain. Two of these ASVs belonging to the families Chitinophagaceae and MLE1-12 were also reduced in both reproductive organs, and the third ASV belonging to the family Sphingomonadaceae, species *Sphingomonas yabuuchiae*, was also reduced in the uterus. The heart showed a decrease in one ASV belonging to the family Veillonellaceae, species *Veillonella dispar*, and increases in two ASVs belonging to the families Pseudomonadaceae and Enterobacteriaceae. Only one ASV belonging to the family Clostridiaceae, species *Clostridium perfringens*, was found to be significantly reduced in the liver, with no ASVs identified as significantly increased. Lastly, only two ASVs were found to be increased in the spleen, belonging to the families Peptostreptococcaceae and Chitinophagaceae, genus *Sediminibacterium*.

Analysis of composition of microbiomes analyses identified very few meaningful differences in bacterial relative abundance between causes of death, but several differences are worthy of remark. We observed an increased relative abundance (>20 log2 fold change relative to other causes of death) of ASVs belonging to the families MLE1-12, Enterobacteriaceae, and Chitinophagaceae among individuals who died of natural causes. Accidental deaths showed no meaningful differences from other manners of death, while death by homicide appeared to be slightly negatively associated (∼5 log2 fold change) with ten unique ASVs belonging to the class Bacilli, primarily in the families Streptococcaceae and Lactobacillaceae. Lastly, death by suicide showed a strong negative association with an ASV in the family Peptostreptococcaceae (>20 log2 fold change relative to other manners of death).

### Association Between PMI and Bacterial Relative Abundances

**FIGURE 4:**
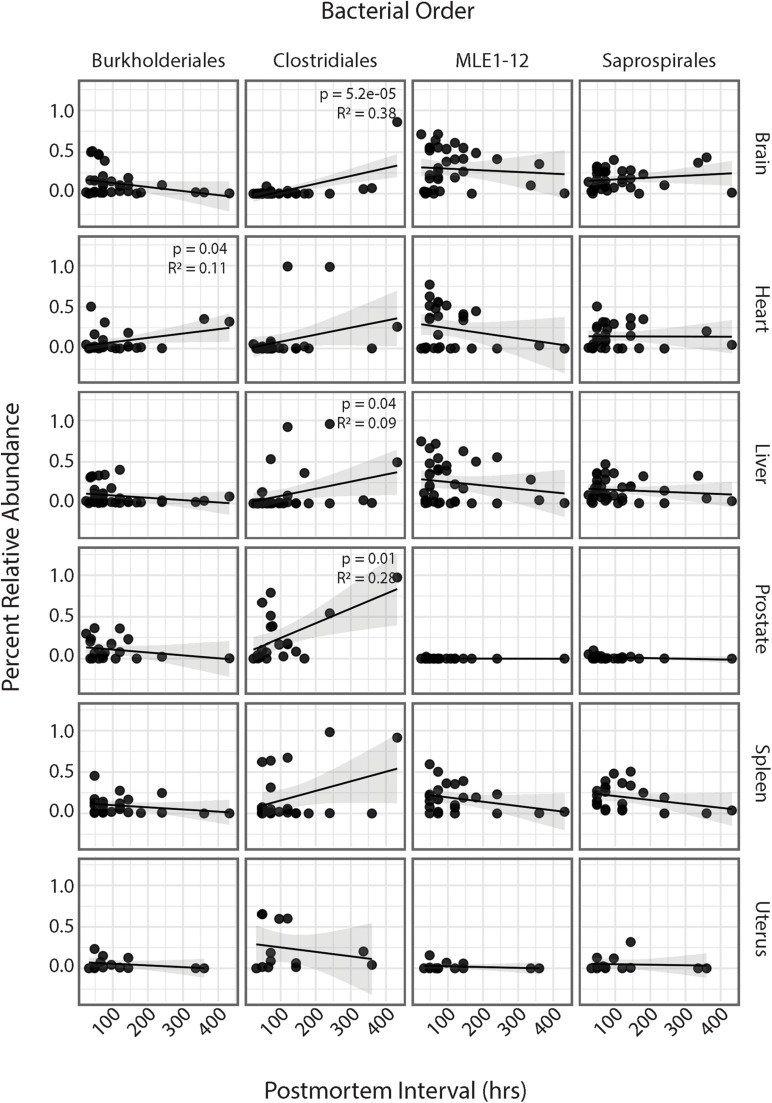
Linear regression of relative abundance of four most abundant bacterial orders by PMI across each organ. Significance values are annotated in top right corner for each regression. Non-significant values not labeled.

Regression analyses of PMI and relative abundances of the four most abundant bacterial orders across all six human organs identified several significant relationships (Figure 4). Within the heart, ASVs belonging to the order Burkholderiales showed a significant increase in relative abundance with increasing PMI (*R*^2^ = 0.11; *p* = 0.04). Among all organs except the uterus, ASVs belonging to the order Clostridiales showed increased relative abundance with increasing PMI, but these trends were significant only for the brain (*R*^2^ = 0.38; *p* = 5.2e-5), liver (*R*^2^ = 0.09; *p* = 0.04), and spleen (*R*^2^ = 0.28; *p* = 0.01). Among the brain, heart, liver, and spleen, ASVs in the order MLE1-12 showed a slight decrease in relative abundance with increasing PMI, but these results were not significant. Lastly, ASVs belonging to the order Saprospirales showed no discernable trends in relation to PMI.

## Discussion

The utilization of genetic (Javan et al., 2020) and microbial data in the context of forensic investigations holds great promise for the field of forensic science (Bell et al., 2018; Oliveira and Amorim, 2018). Identification of taxa that are associated with PMI, specific causes of death, or other traits such as age, sex, and BMI may allow investigators to refine the circumstantial details surrounding the death of an individual. In this study, we found the order Clostridiales and the family Saprospiraceae to be among the most dominant putrefactive taxa in the cadre of bacteria that comprise the cadaver internal organ microbiome. Specifically, brain and spleen samples demonstrated significant increases in relative abundances of Clostridiales, as well as increases observed in heart, liver, and prostate tissues. These results, with the exception of uterus, at various times of death confirmed the Postmortem *Clostridium* Effect (PCE) which points to the observation of Clostridia in internal organ tissues (Javan et al., 2017; Pechal et al., 2018; Burcham et al., 2019; Junkins et al., 2019). Further, we also identify a number of cadaver-specific traits (sex, PMI, BMI, and cause of death) to be associated with microbial alpha and beta diversity, as well as bacterial taxa that are differentially associated with these traits.

Postmortem observations of human reproductive organs have demonstrated that the nulligravid uterus and prostate are the last organs to putrefy during decomposition (Casper, 1861; Scott et al., 2020). The prostate’s anatomy is juxtaposed to the urethra, and it is exposed to microorganisms residing in the urinary tract (Shrestha et al., 2018). Also, the uterus’s anatomy has the greatest content of muscle fibers found in the corpus uteri (Schwalm and Dubrauszky, 1966). There is a limited number of microorganisms (e.g., *Clostridium* spp.) that have collagenolytic proteases essential for the degradation of the collagen coating surrounding the prostate and uterus thereby lending to the resistance to their decay (Suzuki et al., 2006). In the natural order of decomposition, human decay is first visible on the abdominal wall, where gut bacteria, such as coliforms and *Clostridium*, proliferate and decompose hemoglobin into greenish compounds that stain the skin. Decomposition typically begins at the gut area unless the cause of death involves asphyxiation by hanging, inflammation of the protective membranes covering the brain and spinal cord (meningitis), drowning, and sun poisoning due to an increase of blood to the skull. Internal organs (liver, pancreas, spleen), that are in close proximity to the gut will immediately begin to putrefy in a particular natural order of decomposition.

Also of interest in this thanatomicrobiome study is the Saprospirales order. These bacteria did not conform to the stereotypical examples of decomposers. Saprospirae are large, filamentous microorganisms that are isolated from aquatic environments, predominantly marine-associated, but also freshwater and activated sludge. Members in this family breakdown polysaccharides and use the products of hydrolysis for the conversion of nutrients into bacterial biomass (Xia et al., 2008) which are important processes in human putrefaction. The helical gliding strains of this family are also associated with predation of other bacteria producing selective forces that increase their abundance. These bacteria are isolated most frequently from environmental sources; however, the study of the thanatomicrobiome of internal organs is not directly influenced by the same environmental abiotic factors (i.e., pH and temperature) and biotic factors (i.e., insects and scavenger activities) that are encountered by the other environmental samples (Javan et al., 2016). Some studies have shown that internal body sites with restricted access to environmental taxa decay in a relatively normal fashion; however, they decompose more slowly than body sites invaded by taxa from gravesoil and aquatic ecosystems (Preiswerk et al., 2018).

The significant patterns observed in the current study that are associated with cause of death warrant reinforcement from additional investigations to elucidate the origin of these associations. A retrospective study of Italian criminal cases demonstrated that 34% of decedents had no documented or an inaccurate cause of death (Di Vella and Campobasso, 2015). The majority of inaccuracies are results of over-diagnoses of natural deaths, specifically ischemic heart disease, hypertensive cardiovascular disease, and cancer. The risk of misdiagnoses is particularly high in traumatic deaths that do not include blood samples for toxicological testing and/or radiological imaging.

Microbes colonizing organs would decompose them differently depending in the manner of death classification (natural, suicide, homicide, or accident). Host bodies that died as result of natural death (e.g., cancer, cardiovascular disease, diabetes) will generally putrefy more slowly than death due to breaches in the skin (e.g., gunshot wounds, blunt force trauma) that permit entry of environmental bacteria and abiotic and biotic factors (Javan et al., 2019). In a recent study to determine how postmortem microbiome beta dispersion could be an additional forensic tool for predicting cause and manner of death in medicolegal investigations, a surprising finding demonstrated that the beta diversity significantly differed among body sites and causes and manners of death. For example, microorganisms detected in cardiovascular disease cases had significantly increased beta dispersion among all body locations (eyes, ears, nose, rectum, and mouth) (Kaszubinski et al., 2020). Therefore, the increase in microorganisms in cardiovascular disease cases demonstrates dysbiosis in the dead, and that microbial signature variability may be measured through beta-dispersion calculations (e.g., Kruskal–Wallis).

To date, a universal approach to determine the cause of death remains an enigma. There are only a few methods presently used that employ molecular biology to diagnose the cause of death. Ackerman et al. (2001) coined the term “molecular autopsy” to describe the studies of DNA extracted from cadaveric blood and tissue samples to establish the cause of death in autopsy-negative cases. During molecular autopsies, specimens are taken from the cadaver and DNA is extracted using commercially available DNA extraction kits. Sequencing of the extracted DNA is analyzed via traditional Sanger sequencing and high-throughput next-generation platforms to detect disease-causing mutations (Boczek et al., 2012). The sequencing and bioinformatics techniques described in the current study suggest that an increased subject size and varying times of death will provide insights for the utility of this methodology to determine the cause of death.

There are limitations of this methodology which include the paucity of known reference genomes for sequence comparisons (Pechal et al., 2014). Furthermore, despite the efficacy of this novel approach to molecular autopsy, a larger sample size of deceased cases is needed to further account for variation in postmortem microbial communities associated with human decomposition in different habitats.

## Conclusion

The advancement of the thanatomicrobiome as a forensic indicator of the cause of death requires investigation of unknown relationships between human remains, fundamental decomposition processes, and internal microorganisms. There are a variety of abiotic and biotic factors obtained from cadavers that have been investigated as biomarkers to assist forensic pathologists in the determination of the cause of death. Current medicolegal investigative techniques have their merits; but in reality, most of the molecular approaches are limited to the approximation of PMI and the cause of death. Furthermore, the approaches do not provide definitive determinations of the cause of death, which remain an enigma. Future experiments using our thanatos model system that include a larger cohort of samples from both violent and pathological deaths and more internal organs will be necessary to determine the potential impact of the relationship between postmortem microbial succession and the cause of death. Finding a new method which takes the human error out of PMI calculations and determinations of the cause of death would be a milestone in solving questionable criminal cases. Robust statistical models have to be applied to account for the microbial variability demonstrated among different causes of death. Furthermore, in the era of COVID-19, accurate autopsies are crucial in order to determine the cause of death in decedents who test positive for SARS-CoV-2 and to discriminate between those who died *from* COVID-19 and who died *with* COVID-19.

## Data Availability Statement

The datasets presented in this study can be found in online repositories. The names of the repository/repositories and accession number(s) can be found in the article/Supplementary Material. The EBI Accession Number is PRJEB41511.

## Ethics Statement

The study was approved by the Committee for the Protection of Human Subjects, Alabama State University Institutional Review Board (IRB) number 2020100. Methods were in accordance with relevant guidelines and regulations regarding working with cadavers. Written informed consent was obtained from next-of-kin relatives of the cases.

## Author Contributions

GJ and JG designed the study. SV and AO collected the human corpses. GJ and SF extracted the genomic DNA, PCR, and gel electrophoresis the postmortem samples. NG performed the MiSeq sequencing. HL performed the bioinformatic data analysis. HL, GJ, and SF wrote and edited the amnuscript. All authors read and approved the final manuscript.

## Conflict of Interest

The authors declare that the research was conducted in the absence of any commercial or financial relationships that could be construed as a potential conflict of interest.

## Footnotes

1 http://greengenes.lbl.gov

2 http://github.com/hollylutz/CadaverMP

3 http://qiita.ucsd.edu

## Supplementary Material

Supplementary Table 1 Demographic data for each of the 40 cadavers. The cases were collected at the Department of Public Health, Experimental and Forensic Medicine at the University of Pavia in Pavia, Italy.
